# Impact of the Cultivation System and Plant Cultivar on Arbuscular Mycorrhizal Fungi of Spelt (*Triticum aestivum* ssp. *Spelta* L.) in a Short-Term Monoculture

**DOI:** 10.3390/pathogens11080844

**Published:** 2022-07-28

**Authors:** Justyna Bohacz, Teresa Korniłłowicz-Kowalska, Kamila Rybczyńska-Tkaczyk, Sylwia Andruszczak

**Affiliations:** 1 Department of Environmental Microbiology, Faculty of Agrobioengineering, University of Life Sciences in Lublin, Leszczynskiego 7 Street, 20-069 Lublin, Poland; teresa.kornilowicz@onet.pl (T.K.-K.); kamila.rybczynska-tkaczyk@up.lublin.pl (K.R.-T.); 2 Department of Herbology and Plant Cultivation Techniques, Subdepartment of Agricultural Ecology, Faculty of Agrobioengineering, University of Life Sciences in Lublin, Akademicka 13 Street, 20-950 Lublin, Poland; sylwia.andruszczak@up.lublin.pl

**Keywords:** arbuscular mycorrhizal fungi (AMF), number of spores, root colonization, spelt, cultivars, tillage and no-tillage systems

## Abstract

Native communities of arbuscular mycorrhizal fungi (AMF) constitute a natural biofertilization, biocontrol, and bioprotection factor for most agricultural crops, including cereals. The present study investigated the native AMF population in cultivated spelt, i.e., a cereal that has not been analyzed in this respect to date. In particular, the aim of the study was to determine the number of spores and the degree of AMF root colonization in two spelt cultivars (Franckenkorn and Badengold) from a 3-year monoculture grown in two different cultivation systems: conventional tillage and no-tillage systems. The study showed considerable accumulation of AMF spores in the soil (on average 1325 in 100 g of air-dry soil), with a wide range of their numbers, and not a very high degree of endomycorrhizal colonization (on average from 3.0% to 31%). The intensity of AMF growth in the subsequent cultivation years gradually increased and depended on the cultivation system as well as the growth stage and cultivar of the spelt. It was found that both analyzed AMF growth indices in the no-tillage system were positively correlated with each other. Moreover, their values were higher in the no-tillage system than in the conventional system, with statistical significance only for the number of spores. This was mainly observed in the variant with the Franckenkorn cultivar. The effect of the growing season was evident in both cultivation systems and spelt cultivars. It was reflected by intensification of sporulation and mycorrhization of spelt roots by AMF in summer (maturation stage) compared with the spring period (flowering stage).

## 1. Introduction

The root microbiome plays an important role in plant growth and health, as it supports nutrient uptake, enhances tolerance to abiotic stresses, and offers protection against soil pathogens [1,2]. Mycorrhizal fungi are one of the most important components of the root microbiome in terms nutritional and protective functions. In the case of herbaceous plants, including crop species, endomycorrhizal fungi representing arbuscular mycorrhizal fungi (AMF) have the most important functions. Most *Gramineaceae* (including cereals) and *Fabaceae* species are highly mycotrophic plants. In contrast, *Brassicaceae* and *Chaenopodiaceae* are autotrophic plants or are rarely infected by AMF [3]. The arbuscular mycorrhiza comprises obligatory biotrophs from the phylum Glomeromycota. Inside the cortex, they form so-called arbuscules involved in the exchange of nutrients between symbionts and (but not always) so-called vesicles serving storage functions. They form loose mycelia and spores on the outer side of roots [4,5].

The value of arbuscular mycorrhiza as a plant-growth stimulating factor is attributed to their role in the increase in the uptake of poorly labile minerals, mainly phosphorus. The uptake is facilitated by the extraradical mycelium, which enlarges the root absorptive surface, and the ability of these fungi to absorb phosphorus from plant-inaccessible sources [6,7]. A sufficient level of endomycorrhizal root infection contributes to an increase in the uptake of other poorly labile ions, e.g., NH_4_^+^, Zn^2+^, or Cu^2+^ [8,9,10,11]. Changes in plant physiology induced by endomycorrhizal symbiosis, i.e., improved nutrition, alterations in the quantitative and qualitative composition of root exudates, and synthesis of antagonistic substances, improve plant resistance to fungal diseases of the root system [6,10,12]. However, the biocontrol function of AMF depends on the genetically determined sensitivity of a plant species or cultivar to rapid and effective endomycorrhizal colonization of roots [13]. Colonization of roots by AMF increases plant tolerance to drought, which results from, e.g., the possibility of water uptake at low soil water potential and greater soil exploration by the root system [6,14,15]. Arbuscular mycorrhiza also enhances host plant tolerance to soil salinity, acidification [16,17,18], and heavy metals [19].

In dense plant communities, e.g., in meadow sward or cereal swath with interpenetrating root systems, nutrients (especially C, P, and N) flow between plants through the AMF hyphal network connecting the roots [20,21]. AMF also has an impact on the formation of microbiocenotic relationships in the plant rhizosphere (mycorhizosphere) [13], which is influenced by changes in the root secretion process [22]. They stimulate the growth of N_2_-assimilating and orthophosphate-solubilizing bacteria and antagonistic microorganisms serving biocontrol functions [16,22]. The stimulation of saprotrophic fungi with antagonistic abilities such as *Trichoderma* and *Penicillium* by AMF in the plant rhizosphere was reported by Thanoon and Jamiołkowska [23]. AMF also improve the soil structure through enhancement of the stability of soil aggregates by glycoproteins called glomalins produced by these fungi and acting as a binder and a hyphal network penetrating the soil [10,16,24]. The effectiveness of symbiosis, i.e., a process that brings evident benefits to the plant in terms of both nutrition and response to unfavorable environmental conditions and pathogens depends on a large number of factors: the degree of mycotrophicity of the plant, the efficiency of the fungus in ion uptake from the soil, abiotic and biotic factors in the soil environment (in particular, phosphorus content), climatic conditions, and agrotechnical treatments [25,26,27]. In agroecosystems, the density of spores, biodiversity, and root colonization by AMF can change substantially depending on the cultivation system, fertilization, and plant protection products used [28]. As suggested by some authors [29,30], effective symbiosis requires at least 50% root colonization. However, the degree of root colonization by AMF depends on the type of soil, crop species, cultivation system, and soil infection potential [28,31,32,33,34]. The group of edaphic factors that limit endomycorrhizal colonization includes a high level of soil phosphorus, organic C, nitrogen, and strongly acidic or alkaline reaction of the soil [25,35,36]. In turn, soils with low content of phosphorus and organic matter with slightly acidic or neutral reaction are considered favorable for plant mycorrhization [25,37,38,39].

Wheat is one of the crop plants with high susceptibility to mycorrhizal infection [31]. Previous studies of the presence of AMF in the wheat growth environment and mycorrhization of roots in this cereal focused on the common wheat *(Triticum aestivum* ssp. *vulgare* L.) [31,37,38,40,41]. Spelt (*Triticum aestivum* ssp. *spelta* L.), i.e., one of the oldest crops, has been experiencing a renaissance in recent years due to its special nutritional and health-enhancing properties. Research on the root microbiome of this plant has been carried out by Kuźniar et al. 2020 [42], Ratajczak et al. (2020) [43], Salamon et al. (2020) [44], and Korniłłowicz-Kowalska et al. (2022) [45]. However, these studies provide no information on the native AMF population in spelt cultivation. The aim of this study was to determine the number of spores of the native AMF population and the degree of root colonization by these fungi in two spelt cultivars grown in tillage (conventional) and no-tillage (simplified) systems. Taking into account the aim of the study, a three-point research hypothesis was formulated: (1) the cultivation system exerts an impact on the sporulation and colonization of spelt roots by AMF; (2) spelt varieties differ in the density of AMF spores in the soil and the degree of endomycorrhizal colonization of the roots; (3) the dynamics of seasonal changes in the number of spores and root colonization by AMF is associated with the development of the host plant. The study provides data completing our previous investigations [45] on the communities of non-mycorrhizal fungi, rhizosphere soil, and roots of the same spelt cultivars grown in the two systems.

## 2. Results

### 2.1. Number of AMF Spores 

**Figure 1:**
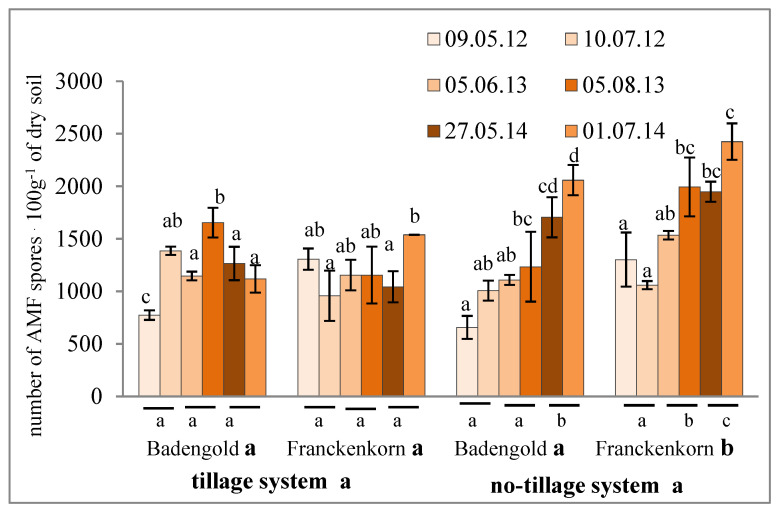
Number of AMF spores in the soil under cultivation of two spelt cultivars (Badengold, Franckenkorn) in the tillage and no-tillage systems; *n* = 4. Explanations: the same letters denote means that do not differ significantly from each other (*p* < 0.05); different letters denote means that differ significantly from each other (*p* < 0.05).

The isolation, separation, and counting demonstrated that the number of AMF spores in the soil samples taken from all four experimental combinations (two spelt cultivars, each in two cultivation systems) was in the range from 648 to 2424 in 100 g of air-dry soil (Figure 1), with the mean number of 1325 spores in the 3-year cultivation period. Spores with a diameter above 50 µm accounted for 82.4–98.3% of the total number (Table 1). The lowest number of spores of each spelt cultivar in both cultivation systems was recorded in the 1st year of the experiment. In both subsequent years, the number of spores increased with statistical significance only in the no-tillage system. This was recorded in the 2nd and 3rd year of cultivation of the Franckenkorn (F) cultivar and in the 3rd year of cultivation of Badengold (B) (Figure 1).

In terms of the differences between the cultivation systems, the mean number of spores of cultivar F was significantly higher in the no-tillage *versus* tillage system (Figure 1) and correlated with these variables (r = 0.5477 *). In turn, the mean numbers of spores in the cultivar B variant in both systems were similar (no statistically significant differences) and were not significantly correlated (r = 0.2847) (Figure 1). The comparison of the number of spores between the cultivars grown in each of these cultivation systems indicated significantly higher abundance in the F than B variant in the no-tillage system, whereas no significant differences were found in the number of fungal spores between these cultivars in the tillage system (Figure 1). Additionally, the numbers of spores in the soil from variants B and F were significantly positively correlated in the no-tillage system (r = 0.8438 *), while no such correlation was found in the tillage system (r = 0.3427).

The impact of the growing season on the sporulation of AMF was evident in the tillage system. This was proved by the significantly higher number of spores in the summer months (July, August) than in the spring period (May, beginning of June) in the 2nd and 3rd cultivation year in the cultivar B variant and in the 3rd year in the case of cultivar F. Similarly, the mean numbers of spores in the no-tillage system were higher in summer than in spring in the case of both cultivars B and F; however, these differences were statistically insignificant (Figure 1).

### 2.2. Degree of Endomycorrhizal Colonization 

**Figure 2:**
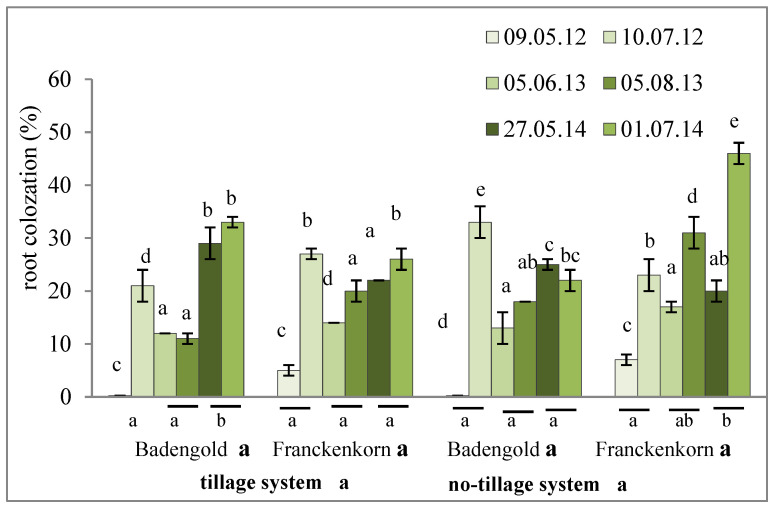
Degree of AMF colonization of Badengold and Franckenkorn spelt roots in the tillage and no-tillage systems; *n* = 4. Explanations as in Figure 3.

The rate of AMF colonization of the spelt root tissues ranged from 0% to 44% (Figure 2). Throughout the study period, the average degree of endomycorrhizal root colonization ranged from 3.0% to 31.25% in both spelt cultivars and both cultivation systems. The lowest degree of spelt root mycorrhization was recorded in the 1st year (on average from 10.5% to 16.5%), whereas the most intensive colonization was noted in the 3rd year (on average from 23.5% to 33.0%). This effect was statistically significant in the F variant in the 2nd and 3rd cultivation years in the no-tillage system and in the B variant in the 3rd year in the tillage system (Figure 2). A comparison of the endomycorrhizal colonization degree between the cultivation systems showed that the mean values of root endomycorrhizal colonization in both spelt cultivars were higher (especially in cultivar F), although insignificantly, in the no-tillage than tillage system. The differences in the root mycorrhization degree between cultivars B and F in each of the cultivation systems were statistically insignificant as well. The trend was, however, similar, i.e., the AMF colonization of cultivar F was more effective than in the case of cultivar B (Figure 2). Moreover, in the no-tillage system, the degree of colonization of the roots in cultivar B was significantly positively correlated with the degree of colonization of the roots in cultivar F (r = 0.4861 *). No such relationship was found in the case of the tillage system. It was found, however, that the degree of mycorrhization of the roots in cultivar F in the tillage system was correlated with the degree of colonization of this cultivar grown in the non-tillage system (r = 0.7413 *).

In both cultivation systems, the growing season was found to exert an impact on root mycorrhization, as evidenced by the significantly higher degree of root colonization by AMF in summer than in spring. This was clearly visible in the case of cultivar F, where the effect was detected in each experimental year. In the case of cultivar B, significantly higher root mycorrhization in summer than in spring was recorded only in the 1st year of the experiment in both cultivation systems (Figure 2).

### 2.3. Relationships between the Total Number of Spores and AMF Root Colonization (Correlation Analysis) in Relation to Sampling Dates (PCA Analysis)

It was shown that the total mycorrhization of the roots of both spelt cultivars in the no-tillage system was significantly positively correlated with the number of AMF spores in the soil under these cultivars (r = 0.6361 *). No such relationship was found in the tillage system (r = 0.2919). The analysis of both cultivation systems revealed a significant positive correlation between the degree of colonization and the number of spores only for cultivar F (r = 0.5640 *), whereas no such correlation was found for cultivar B (r = 0.1273). The analysis of each cultivar and cultivation system separately showed a significant correlation between the AMF activity indicators only in the case of cultivar F in the no-tillage system (r = 0.7376 *). The correlation for cultivar B was insignificant (r = 0.4617). No significant relationship was determined for each of these cultivars in the tillage system. The negative (insignificant) values of the correlation coefficient (cultivar B: r = −0.3908; cultivar F: r = −0.1127) may even suggest a potential inverse relationship between the number of spores and the degree of spelt root colonization in this system.

**Figure 3:**
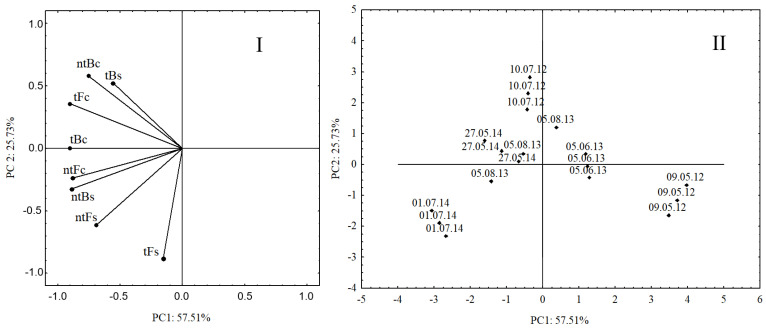
PCA loading plot (**I**) and score plot (**II**) for the results of the number of spores (s) and the degree of colonization (c) in the tillage (t) and no-tillage (nt) systems.

The PCA model (Figure 3) based on the degree of endomycorrhizal colonization of spelt roots, the number of AMF spores in both cultivars in the two cultivation systems, and the different analysis dates explains 83% of the variability of the two main components (PC1—57.51% and PC2—25.72%, respectively). The model shows that the early terms, i.e., May 2012 and June 2013, did not support the processes of AMF sporulation (number of spores) and mycorrhization (degree of root colonization). This is evidenced by the same location of the dots on the score plot relative to the variables on the loading plot. In turn, the location of the dots on the score plot clearly indicates that the last analysis date, i.e., July 2014, is correlated with the number of spores in cultivars B and F in the no-tillage system and the colonization of the cultivar F roots in this system. Moreover, Figure 3 shows visible changes in both analyzed parameters mainly in cultivar B in both cultivation systems in July 2012, August 2013, and May 2014. In the case of cultivar F, significance was found only in the case of AMF colonization of roots in the tillage system.

## 3. Discussion

Previous studies on the presence of AMF in various soils in Poland under the cultivation of cereal plants reported a very large range of fungal spore density, i.e., from 0 to 3230 in 100 g of air-dry soil [40,46,47]. Księżniak and Kobus (1998) [40] analyzed many different types of soil and samples of mixed rendzina soil collected in different locations of cultivation of four basic cereals (wheat, barley, rye, and oats) and detected the presence of 910-3230 fungal spores in 100 g of air-dry soil. The highest numbers of AMF spores were recorded in samples collected from soil under common wheat (*Triticum aestivum* ssp. *vulgare* L.) cultivation. The present study showed that the number of AMF spores in the mixed rendzina under the cultivation of spelt (*Triticum aestivum* ssp. *spelta* L.) was 25–28% lower (from 658 to 2424 spores/100 g air-dry soil) than the data reported by Księżniak and Kobus (1998) [40] for the common wheat. These differences are significant but not very high, bearing in mind that different wheat species were analyzed. Given the order of the density of AMF spores in cereal plants determined by the authors cited above, i.e., wheat > rye > oats > barley, the spore density in spelt roots ranks second after the common wheat. Moreover, the very high percentage (on average 82% to 98%) of spores with a diameter larger than 50 µm indicates high viability of the native AMF population under the spelt cultivation, which is in agreement with the findings reported by Księżniak and Kobus (1998) [40].

The present study demonstrated that the number of AMF spores in the 3-year spelt monoculture cultivation increased from year to year. It was calculated that the increase in the number of spores (taking into account all four experimental combinations) in the 2nd and 3rd cultivation years ranged from 10.0% to 12.6%. Significantly higher values were obtained only in the no-tillage system, where they ranged from approx. 50% in the 2nd year to 80% in the 3rd year of cultivation of the Franckenkorn cultivar (F) and 126% in the 3rd year of cultivation of the Badengold cultivar (B). A very slight increase in the number of spores in the two subsequent years of spelt cultivation was found in the tillage system (slightly above 11%). A lower number of spores under cereal cultivation (common wheat and oats) in conventional tillage *versus* no-tillage system was previously reported by Castillo et al. (2006) [48] in analyses of AMF propagules in a short 2-year-course crop rotation system.

The more intensive sporulation of AMF in the simplified cultivation system (no-tillage system) compared with the conventional one (tillage) was mainly related to the maintained continuity of the network of extraradical hyphae, which are mostly involved in the production of spores. In the conventional system, it was not possible to maintain the extra-matrix mycelium continuity due to the plowing treatments. This was emphasized by Castillo et al. (2006) [48] and Usuki et al. (2007) [33], who reported that tillage in cultivated soils caused fragmentation of the extensive networks of AMF hyphae and limited sporulation. Furthermore, as indicated by Kabir (2005) [49] and Sheng et al. (2013) [50], tillage dilutes AMF propagules. This is caused by the displacement of spores into the soil (<15 cm) from its surface layer, where they are produced [40]. In no-tillage fields, the mycorrhizal system is more stable because it is not exposed to the destructive effects of plowing and is intact in winter [50]. As reported by Usuki et al. (2007) [33], tillage is a major stress factor leading to a decline in the potential AMF inoculum in the soil. Additionally, as highlighted by Roger-Estrade et al. (2010) [24], no-tillage fields provide plants with better nutrition than the tillage system, as their highly branched root system can penetrate the soil more extensively with its intact extraradical mycelium network. This, in turn, has an impact on the growth of the mycobiont, as the improvement of plant nutrition contributes to a greater flow of assimilates, i.e., a source of C and energy for these obligatory biotrophs, to the roots [51]. 

The results of the present study also indicate more effective AMF mycorrhization of spelt roots in the no-tillage system. Undoubtedly, this is associated with the greater infection potential of the soil in this system, as evidenced by the higher number of spores and the positive correlation between the degree of endomycorrhizal colonization of spelt roots and the number of spores as well as the absence of such a relationship in the conventional system (tillage). As reported by Kabir (2005) [49], extraradical AMF hyphae that overwinter in no-tillage fields remain viable as an inoculum in spring. An intact AMF filament network left after a previous no-tillage growing season provides AMF inoculum, which enhances the mycorrhizal colonization of roots in the consecutive growing season [48]. In turn, disturbances in these hyphae result in limited mycorrhizal colonization of roots, likewise the tillage system [40].

The present investigations indicate a relatively low degree (maximum 44%) of mycorrhization of spelt roots. In contrast, the degree of endomycorrhizal colonization of winter wheat (common wheat) by native populations of AMF in various soils in Poland studied by Księżniak and Kobus (1997) [31] ranged from 48% to 90%. As suggested by Błaszkowski (1993) [52], the relatively low level of endomycorrhizal colonization of mycotrophic plant roots may indicate hardly favorable conditions for AMF in the plant growth environment. As emphasized by Gosling et al. (2010) [53], many different agricultural practices, including fertilization, are harmful to AMF. As a result, in many agricultural soils, AMF communities are impoverished, which results in a low level of AMF colonization. The strongest inhibitory effect on the establishment of the symbiosis between AMF and plants is exerted by a high concentration of phosphorus in the soil. This was confirmed in studies on agricultural soil conducted by Jansa et al. (2009) [54], who reported a negative correlation between the phosphorus content and the degree of endomycorrhizal colonization of roots with an inconsiderable impact of other soil parameters, e.g., pH. Given the high concentration of phosphorus (40.64 mg·g^−1^ soil) in the mixed rendzina samples analyzed in this study, it seems reasonable to claim that this parameter was one of the most important causes of the relatively low degree of AMF colonization of spelt roots.

As shown in the present study, the average degree of endomycorrhizal colonization of roots was generally higher (but not always statistically significant) in the Franckenkorn spelt cultivar than in Badengold, as in the case of AMF sporulation. Differences in the susceptibility to endomycorrhizal symbiosis of not only species but also plant cultivars and, consequently, differences in growth and other physiological processes of the host plant were reported by Allen et al. (1995) [27], Wu et al. (2005) [55], Nasrullah et al. (2010) [38], Mobasser et al. (2012) [56], and Ratajczak et al. (2020) [43]. In their recent study on three AMF-inoculated spelt cultivars grown in drought conditions, Ratajczak et al. (2020) [43] showed that the plants differed in photosynthetic activity, aboveground biomass, and root dry weight and length. The authors noted that the increase in the root length in some AMF-inoculated spelt cultivars contributed to substantial enlargement of the root system compared with other cultivars. A similar phenomenon may have occurred in the spelt cultivars analyzed in the present study. It is possible that the Franckenkorn cultivar with the established endomycorrhizal symbiosis was characterized by greater expansion of the root system than in the case of the Badengold cultivar. This may have had an impact not only on the mycorrhization of roots but also on the mycobiota sporulation. Given the data reported by Ratajczak et al. (2020) [43], the differences in the mean number of fungal spores between the Franckenkorn and Badengold cultivars may have been associated with the differences in the extraradical mycelium size. From the point of view of root mycorrhization, it seems that a more extensive fibrous root system (as in cereals) facilitates more effective root colonization and formation of a denser network of extraradical AMF hyphae than a less developed root system in these plants. This suggestion was supported by Andruszczak et al. (2012) [57], who showed that the Franckenkorn variety has a larger leaf surface, which enhances the photosynthetic activity and growth in this cultivar. This finding also applies to the root system. This variety is also characterized by a more horizontal arrangement of leaves, which contributes to better shading of the soil and greater limitation of weed infestation than in the case of the Badengold variety [57]. This, in turn, increases the ability of the roots to penetrate the soil and results in better availability of nutrients in the soil in the case of Badengold. Another cause of the differences in the density of spores and the degree of endomycorrhizal colonization of the spelt cultivars may be related to differences in the quantitative and qualitative composition of root exudates. This assumption is supported by the information provided by Iannucci et al. (2017) [58]. The researchers showed that the composition of metabolites secreted into the rhizosphere was associated with the tetraploid wheat genotype (*Triticum turgidum* L.). Moreover, they showed diverse types and magnitudes of the effects of these metabolites on communities of rhizosphere microorganisms. Since root exudates have an impact on the sporulation and mycorrhization of wheat roots by AMF [38], we suppose that the differences in the root secretion between the spelt Franckenkorn and Badengold cultivars may have been responsible for the differences in endomycorrhizal sporulation and colonization. However, these are only assumptions that need to be confirmed for the spelt cultivars analyzed in the present study.

The present study clearly showed that both the number of spores and the degree of AMF colonization of spelt roots underwent large seasonal fluctuations. This was reflected in the more intense AMF sporulation and mycorrhization of the roots in summer than in spring. A similar seasonal dynamic of both these indicators of AMF growth in the climatic conditions of Central and Eastern Poland was reported by Korniłłowicz-Kowalska et al. (2020) [59] in a study of meadow plant communities (grass and clover) growing on organic soil. Intensification of AMF sporulation and stimulation of the growth of arbuscular mycorrhiza in summer *versus* the beginning of the growing season were also observed by Panwar and Tarafdar (2006) [60] and Panwar et al. (2011) [37]. As reported by Rodriguez-Echeverria et al. (2008) [61], the seasonal changes in AMF abundance and endomycorrhizal colonization of plant roots are correlated with the stage of host plant development. The association between AMF mycorrhization of roots and host plant phenology was also reported by Li et al. (2005) [62], who investigated the seasonality of occurrence of arbuscular fungi. The weaker growth of AMF observed in the present study in the spring period (spelt flowering in May and early June) may have been caused by the weaker root growth, in contrast to the summer period (spelt maturation stage in July and August). This suggestion is supported by the results reported by Titus and Leps (2000) [63], who observed slower root growth in plants with established endomycorrhizal symbiosis during the flowering period. Furthermore, as demonstrated by Rodriquez-Echeverria et al. (2008) [61], AMF sporulation is inhibited during plant flowering as well, which is associated with the high demand for soil nutrients, e.g., phosphorus, during this plant development stage. AMF spores exhibit lower biodiversity during flowering (spring), compared with the grain filling stage (summer). This was demonstrated by Schalamuk et al. (2006) [28] in a study of spring wheat in no-tillage and conventional tillage systems. A similar phenomenon, which was reflected in the density of spores and the colonization of the plant roots, may have occurred in the analyzed spelt cultivation systems.

To summarize the results, it is worth noting that the role of endomycorrhiza as a specific biological spelt fertilizer, in particular providing phosphorus with simultaneous fertilization with this element, may be insignificant. This observation is related to the fact that the phosphorus uptake by the extra-root mycelium of AMF is enhanced mainly at low levels of this element in the soil, increasing the photosynthesis process and promoting plant growth [4,10]. In turn, the biocontrol function of AMF may be more important for spelt plants. The colonization of root tissues (primary cortex) by the mycobiont is known to activate plant defense mechanisms, preventing infection of roots by phytopathogenic microorganisms such as rot fungi, including *Fusarium* [4,10]. This genus was abundant in the spelt root zone in both cultivation systems analyzed in the present study, but caused no visible disease symptoms in the plants [45].

## 4. Materials and Methods

### 4.1. Characteristics of the Experimental Object

The investigations were carried out in a model field experiment established on the Experimental Farm in Bezek near Chełm (51°19′ N, 23°25′ E; eastern Poland) by the research team from the Department of Agricultural Ecology, University of Life Sciences in Lublin. The experiment was conducted in the growing seasons 2011–2012 and 2013–2014. The experiment was established on mixed rendzina (Rendzic Phaeozem-WRB 2006) [64] formed of chalk rock with a granulometric composition of medium clay. The soil was characterized by alkaline pH (pH = 7.35), high contents of total N (3.05 g·kg^−1^), phosphorus (406.4 mg·kg^−1^), and potassium (194.7 mg·kg^−1^), and very low content of magnesium (17.9 mg·kg^−1^).

The two-factor experiment was established in three replicates using the split-plot method with a plot size of 15 m^2^. Two conventional (tillage) and simplified (no-tillage) cultivation systems were the first-order factor. Two winter spelt cultivars: Badengold (B) and Franckenkorn (F) were the second-order factor. The spelt was cultivated in a short-term monoculture. Spelt spikelets (350 kg·ha^−1^) were sown in mid-October 2011. The following mineral fertilization scheme was applied: N-60, P-26.2, and K-83 (in kg of pure component ha^−1^).

According to preliminary studies, the varieties selected for the experiment are well-adapted to the agroecological conditions of eastern Poland and are characterized by satisfactory yield and high nutritional quality of grain [65]. Although these varieties exhibit high genetic similarity [66], they differ in yield-related biometric traits and the chemical composition of grain. In terms of morphology, the cultivars differ mainly in ear productivity (grain number per ear, grain weight per ear), ear length, thousand-grain weight, number of ears formed by a plant, and plant height. In addition, they differ in the content of macro and micronutrients in the grain as well as the content of protein, gluten, starch, amino acids, and lutein [65,67,68]. In addition, the Badengold and Franckenkorn cultivars have a distinctly differentiating effect on the biodiversity of agrophytocoenoses. Greater species diversity of segetal flora was observed in the case of the variety Franckenkorn, than in Badengold. This variety also exhibits a higher ability to compete with weeds [57,69].

### 4.2. Meteorological Conditions

**Figure 4:**
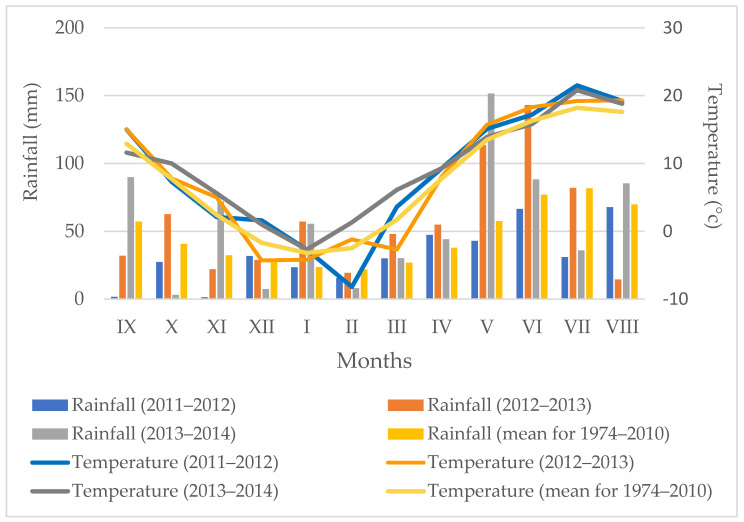
Average air temperature and total precipitation during the growing seasons.

Figure 4 shows the air temperature and precipitation recorded during the field study. The average air temperatures in the three growing seasons, 2011–2012, 2012–2013, and 2013–2014, were generally higher than the long-term average. The greatest differences from the long-term norm were noted in March–September 2012, May–August 2013, and February–April 2014 (Figure 4).

In comparison with the long-term average, the precipitation sums were lower in the 2011–2012 growing season and higher in 2012–2013 and 2013–2014. In the 2011–2012 season, the precipitation rates were on average 30% lower than the long-term norm. The highest humidity shortage was recorded from September to November 2011 and from May to July 2012. The rainfall sums in the 2nd year of the experiment increased on average by 22% in relation to the multiannual average. A particularly high increase in air humidity by 98% and 86% of the long-term norm was recorded in May and June 2013, respectively. In the 3rd year of the experiment, the precipitation sum exceeded the multiannual value (except for October–December 2013 and February–July 2014), and a record increase in precipitation was noted in May 2014 (2.5 times higher than the long-term average) (Figure 4).

### 4.3. Preparation of Research Material

The research material included soil and plant roots collected twice at the spelt flowering (May or June) and maturation (July, August) stages in each year of the 3-year experiment (2012–2014). Samples of soil with plant roots were taken from a depth of 0–20 cm. From each of the three replicates of the experimental combinations, 5–10 samples were collected and mixed to obtain a combined representative sample. In the laboratory, the plant roots were separated from the fresh sample by gentle shaking, leaving a small amount of soil as a protective layer. The combined root samples were placed in string bags and frozen at −18 °C until the assessment of the degree of root colonization by arbuscular mycorrhizal fungi (AMF). Fresh soil that was left after the separation of the roots was sieved through 2 mm mesh sieves. Next, the soil was dried at room temperature (20 ± 2 °C) to air-dry weight. The samples were used for determination of the number of AMF spores. 

### 4.4. Determination of the Number of AMF Spores

The number of AMF spores in the soil samples was determined as in Allen et al. (1979) [70] with modifications proposed by Księżniak and Kobus (1998) [40]. The 50 g air-dry soil samples prepared for the analysis were mixed with 50 g of sand with a grain diameter above 400 µm and suspended in 100 cm^3^ of distilled water in 300 cm^3^ Erlenmeyer flasks. The mixture was shaken at 200 rpm for 4 h and centrifuged at 2000 rpm for 10 min. The supernatant was discarded, and the centrifuged material was suspended in a sucrose solution with 2% sodium polyphosphate containing 684.6 g of sucrose and 10 g of Calgon per 1 dm^3^ of distilled H_2_O. The suspension was centrifuged again at 2000 rpm for 10 min. The spore-containing supernatant was filtered through a set of polyester monofilament sieves (Tetko Inc., New York, NY, USA) with a mesh size of 400, 150, 100, 75, 51, and 20 µm. Each fraction was filtered on a separate acetate filter with 1 × 1 cm^2^ sectors and the spores were counted using a 15–100× stereoscopic microscope equipped with a Camedia 5060 Kide Zoom digital camera. The numbers of spores from each fraction were summed up. Each soil sample was analyzed in duplicate. The results were expressed as an arithmetic mean of two determinations based on 100 g of air-dry soil.

### 4.5. Determination of the Degree of Root Colonization by AMF

The degree of endomycorrhizal colonization of spelt roots was determined using a microscope after staining the root samples with trypan blue as in Philips and Hayman (1970) [71]. After thawing at room temperature, the root samples were gently washed with running water on a 400 µm mesh sieve (Tetko Inc., New York, NY, USA) to remove soil. The roots were cut into 5 mm pieces and left in water for 5 min. The hydrated root samples were heated in 10% KOH at 90 °C for 30 min to decolorize the tissues. Next, they were washed with distilled H_2_O and neutralized in 10% HCl for 1 h. The roots were hot-stained (90 °C, 15 min) with trypan blue in lactoglycerol. The staining mixture contained (g·dcm^−3^) glycerol-0.5, lactic acid-0.25, trypan blue-0.5, and distilled H_2_O-0.25 [72]. Double microscopic slides were prepared from each root sample using polyvinyl-lacto-glycerol (PVLG): polyvinyl alcohol-8.33, lactic acid-0.05, glycerin-0.005, and H_2_O-0.05 g·dcm^−3^ [73,74]. The endomycorrhizal colonization of the roots was assessed with the use of a Nikon Labophot-2 light microscope at a magnification of 40–1000×. The observations were carried out in 50 separate fields of view for each preparation at a magnification of 100×. Arbuscules, vesicles, or hyphae of endomycorrhizal fungi in the root tissue were observed. The results were reported as % of colonization calculated with the following formula: number of colonized fragments/50 root fragments × 100. Both preparations were viewed twice and the mean percentage of root colonization by AMF was calculated.

### 4.6. Statistical Analyses

A multivariate analysis of variance (ANOVA) was used to identify significant differences in the number of spores in the soil and the root mycorrhization between the spelt cultivars (Badengold-B, Franckenkorn-F) and cultivation systems (tillage, no-tillage). It was followed by a Tukey HSD post-hoc test at the significance level α = 0.05. The experimental combinations: tillage system, cultivar B (tB); tillage system, cultivar F (tF); no-tillage system, cultivar B (ntB); no-tillage system, cultivar F (ntF) and the sampling dates: 9.05 and 10.07. 2012; 05.06 and 05.08. 2012; 27.05 and 1.07. 2014, were compared.

To establish the relationship between the number of AMF spores and the degree of mycorrhization of the spelt roots within each cultivation system and between these systems, a correlation analysis was performed with calculation of Pearson correlation coefficients (r) at the significance level α = 0.05. Additionally, correlation coefficients (α = 0.05) were calculated for the number of spores in relation to the cultivar and cultivation system and for the root mycorrhization degree in relation to each of these factors.

Next, the Principal Component Analysis (PCA) was used for accurate determination of the effect of the sampling dates on the number of AMF spores and the degree of endomycorrhizal colonization of the roots of the Badengold (B) and Franckenkorn (F) spelt cultivars in the tillage (t) and no-tillage (nt) systems. The results of the analysis are presented in a loading plot (I) and a score plot (II). The loading plot shows the influence of the variables, i.e., the degree of root colonization, two spelt cultivars, and two cultivation systems, on individual principal components and displays the correlation structure of the variables. The score plot is a map of observations (date of analysis) showing how they are situated with respect to each other based on the variables in the loading plot [75]. Two plots help to analyze the correlation between the observations and the variables. The score plot and the loading plot should be interpreted together [75]. The closer the observation in the score plot (date of analysis) is located relative to the variables (spelt root colonization degree, two spelt cultivars, and two cultivation systems) in the loading plot (i.e., they are in the same part of the loading plot), the higher the impact of the observations on the variables is.

## 5. Conclusions

In conclusion, the present results indicate potentially high infectious activity of the AMF community is rendzina soil with cultivation of spelt, as evidenced by the large abundance and the high proportion of viable spores. However, this is clearly reflected in plant mycorrhization only in the no-tillage system, as evidenced by the significant positive correlation between the number of spores and the degree of spelt root colonization in this system. In turn, the tillage system limits the AMF occurrence and colonization of spelt roots. In this regard, the selection of appropriate cultivars is very important. As demonstrated, only one of the two spelt cultivars analyzed in this study exhibited greater susceptibility to endomycorrhizal symbiosis.

## Figures and Tables

**Table 1:** Proportion of AMF spores with ø > 50 µm in the total number of spores in 100 g of air-dry soil (in %).

Term	Tillage System			No-Tillage System		
	Cultivar Badengold	Cultivar Franckenkorn	Means for Both Varieties	Cultivar Badengold	Cultivar Franckenkorn	Means for Both Varieties
2012						
9.05.	96.08 (736) *	90.70 (1186)	92.70 (961)	87.69 (577)	89.78 (1169)	89.08 (873)
10.07.	92.18 (1277)	90.60 (868)	91.51 (1073)	91.96 (927)	99.87 (995)	92.94 (961)
Means 2012	93.60 (1007)	90.7 (10.37)	92.0 (1017)	90.28 (752)	91.61 (1082)	91.06 (917)
2013						
05.06.	96.25 (1103)	90.83 (1050)	93.54 (1077)	92.07 (1022)	86.90 1333)	89.11 (1178)
05.08.	95.89 (1589)	89.35 (1033)	93.20 (1310)	97.97 (1209)	87.26 (1740)	91.38 (1475)
Means 2013	96.07 (1345)	90.13 (1042)	93.40 (1194)	95.22 (1116)	87.13 1537)	90.39 (1327)
2014						
27.05.	98.33 (1244)	96.55 (1008)	97.49 (1126)	90.03 (1536)	96.80 (1812)	93.57 (1674)
01.07.	86.04 (962)	89.46 (1376)	88.02 (1169)	82.36 (1695)	83.42 (2022)	82.95 (1859)
Means 2014	92.53 (1103)	92.33 (1192)	92.43 (1148)	85.87 (1616)	90.16 (1917)	87.82 (1767)

Explanations: ( ) * mean number of spores with ø > 50 µm.

## Data Availability

Not applicable.
